# Perfusion index assessment during transition period of newborns: an observational study

**DOI:** 10.1186/s12887-016-0701-z

**Published:** 2016-10-07

**Authors:** Sezin Unal, Ebru Ergenekon, Selma Aktas, Serdar Beken, Nilgun Altuntas, Ebru Kazanci, Ferit Kulali, Ibrahim M. Hirfanoglu, Esra Onal, Canan Turkyilmaz, Esin Koc, Yildiz Atalay

**Affiliations:** 1 Division of Neonatology, Department of Pediatrics, Gazi University Hospital, Ankara, Turkey; 2 Department of Neonatology, Etlik Zubeyde Hanım Women’s Health Teaching and Research Hospital, Yeni Etlik Caddesi, 06010 Etlik, Ankara Turkey

**Keywords:** Peripheral perfusion, Perfusion index, Delayed transitional period, Transient tachypnea of newborn, Neonate

## Abstract

**Background:**

Perfusion index (PI) is becoming a part of clinical practice in neonatology to monitor peripheral perfusion noninvasively. Hemodynamic and respiratory changes occur in newborns during the transition period after birth in which peripheral perfusion may be affected. Tachypnea is a frequent symptom during this period. While some tachypneic newborns get well in less than 6 h and diagnosed as “delayed transition”, others get admitted to intensive care unit which transient tachypnea of newborn (TTN) being the most common diagnosis among them. We aimed to compare PI of neonates with TTN and delayed transition with controls, and assess its value on discrimination of delayed transition and TTN.

**Methods:**

Neonates with gestational age between 37 and 40 weeks who were born with elective caesarian section were included. Eligible neonates were monitored with Masimo Set Radical7 pulse-oximeter (Masimo Corp., Irvine, CA, USA). Postductal PI, oxygen saturation and heart rate were manually recorded every 10 s for 3 min for two defined time periods as 10^th^ minute and 1^st^ hour. Axillary temperature were also recorded. Newborn infants were grouped as control, delayed transition, and TTN.

**Results:**

Forty-nine tachypneic (TTN; 21, delayed transition; 28) and 30 healthy neonates completed the study. PI values were similar between three groups at both periods. There were no correlation between PI and respiratory rate, heart rate, and temperature.

**Conclusion:**

PI assessment in maternity unit does not discriminate TTN from delayed transitional period in newborns which may indicate that peripheral perfusion is not severely affected in either condition.

## Background

The transition from fetal to extrauterine life requires multiple rapid organ adaptations. The clearance of fetal lung fluid, surfactant secretion, and the onset of steady breathing occurs during pulmonary adaptation, and changes in blood flow, increase of cardiac output and pulmonary vasodilation take place for cardiovascular transition [1]. During transitional period newborns’ heart rate and oxygen saturations are being continuously observed for several years, whereas peripheral perfusion is not clearly known. Perfusion index (PI) which is calculated as the ratio of pulsatile signal of arterial blood flow to signals from static blood flow, skin, and other tissues which are referred as non-pulsatile signal, is an easy way of monitoring of peripheral perfusion [2]. Studies that assessed PI values during transitional period showed that PI values were highly variable immediately after birth [3, 4], were not associated with mode of delivery [3, 5], and low PI values may predict histologic chorioamnionitis [6].

The transient tachypnea of newborn (TTN) and delayed transition are related issues, however they differ in severity and duration. The TTN which is seen in 5.7–9.7 % of term newborns during the transitional period [7, 8], accounts for the majority of respiratory morbidities requiring neonatal intensive care unit (NICU) admission in term infants, and is a diagnosis of exclusion [9]. It is characterized by pulmonary edema resulting from delayed resorption and clearance of fetal alveolar fluid [10]. Newborns with TTN classically present with increased work of breathing that results in compensatory tachypnea (respiratory rate >60/min) within the first 2 h of delivery. Other respiratory signs include nasal flaring, intercostal and subcostal retractions, and expiratory grunting. Symptoms generally resolve within 12 to 24 h, but may persist for as long as 72 h in severe cases. If respiratory distress in these neonates resolves within 6 h of birth, it is called “delayed transition” [11].

The extracellular volume, pulmonary arterial pressure and N-terminal pro B natriuteric peptide were shown to increase in TTN which all may contribute on mild cardiac systemic dysfunction [12, 13]. Cardiac output of left ventricle is a predictor of tissue perfusion and PI [14]. Therefore peripheral perfusion of neonates with TTN may be affected more when compared with neonates with delayed transition.

It is known in clinical practice that some tachypneic neonates who are admitted to NICU, actually have delayed transition and become clinically stable very soon after admission. Unnecessary admission of neonates were emphasized by Harrison and Goodman as the trend of higher admission rates of higher birth weighted infants and the possibility of overuse of medications to those [15]. There is no recent guideline about the duration for observation of neonates with tachypnea without admission except a previous suggestion of “2 h rule” [16]. This emphasizes the importance of discriminating the tachypneic neonates from who will have delayed transition to avoid unnecessary admissions.

We aimed to compare peripheral perfusion of the tachypneic neonates during first one postnatal hour with healthy newborns by using PI and assess its value on discrimination of delayed transition and TTN to determine if PI assessment in maternity unit would be useful to identify which tachypneic neonates will need NICU admission.

## Methods

The study was conducted prospectively in maternity unit of Gazi University Hospital, Division of Neonatology in Ankara, Turkey. The “Gazi University Medical Faculty Ethics Committee” approved the study. Written informed consents were obtained from the parents of the newborns.

Early-term (gestational age; 37 ^0/7^–38 ^6/7^ weeks) and full term (39 ^0/7^–40 ^6/7^) neonates born with elective caesarian section (C/S) were considered to be eligible if there were no sign of non-reassuring fetal status on fetal nonstress test or biophysical profile, maternal clinical chorioamnionitis, onset of labor or premature rupture of membranes [17, 18]. The eligible newborns were included if they were appropriate for gestational age according to national data [19]. Neonates with meconium stained amniotic fluid, breech presentation, resuscitation including positive pressure ventilation, Apgar score <7 at 5th min, and congenital anomalies involving at least one organ system were excluded.

The included neonates were monitored with Masimo Set Radical 7 pulse-oximeter (Masimo Corp., Irvine, CA, USA). Postductal PI, pulse oximetry oxygen saturation (SpO2), and heart rate (HR) were manually recorded every 10 s for 3 min in two defined time periods being the first at 10^th^ minute of life and the second at the 1^st^ hour of life. The measurements were recorded by a clinical neonatology fellow during steady state of newborn infant. Probe position was ensured and plethysmography pulse wave was confirmed to be artifact free before record. If newborn infants cried or moved vigorously we repeated measurements after a steady state was ensured. Axillary temperature, respiratory rates (RR), capillary refill time were evaluated at 10^th^ minute and at 1^st^ hour. The enrolled neonates were conducted into three groups at the end of sixth hour of life; as “control” if the neonate had normal RR through that time, as “delayed transition” if the neonate had tachypnea for less than 6 h and was not admitted to NICU, and as “TTN” if the neonate had been tachypneic for more than 6 h, admitted to NICU, and tachypnea resolved before the end of fifth day. Neonates who were grouped as TTN were included in the analyses if no clinical or laboratory signs owing to sepsis, pneumonia (parenchymal infiltration in the evidence of infection in blood cell count with maternal history of infection), air leaks, polycythemia, and congenital pulmonary or cardiovascular diseases existed. Management of the neonates were also recorded.

Statistical analyses were performed using SPSS software version 15 (SPSS, Chicago IL, USA). Fit to normal distribution of the variables were investigated using visual (histograms, probability plots) and analytical methods (Kolmogorov-Simirnov/Shapiro-Wilk’s test). Analyses were presented as n (%) or median (interquartile range) where appropriate. The PI values lower than 0.7, 1.0, and 1.24 which are predefined cut-off values for left ventricle outflow obstruction and critical illness were identified, and were compared between three groups if available [20, 21]. Kruskal-Wallis test with Bonferonni correction was conducted to compare the parameters between three groups (control, delayed transition, and TTN groups); *p* < 0.017 was considered for statistical significance. Wilcoxon test was used to compare pairwise measurements of PI within each group. Associations between PI, HR, RR, and temperature were investigated with Spearman test to calculate correlation coefficient and their significance. A *p*-value less than 0.05 was considered to show a statistically significant result.

We did a power analysis (two tailed) to detect a change of 30 % at PI values between groups with 80 % power and α of 0.05. Based on this, 19 neonates in each group were required.

## Results

In four months period 456 deliveries occurred which 111 of them were eligible. There had been 32 missing newborn infants due to unavailability of neonatology fellow who would perform PI monitoring and data recording; at the end 79 neonates completed the study. The study included 21 neonates with TTN, 28 neonates with delayed transition, and 30 neonates with normal respiratory rates (controls), all born after elective C/S. Birth weight of newborns with delayed transition were significantly higher than controls but similar to neonates with TTN. Although ratio of early-term neonates were comparable in three groups, gestational age was found to be higher in neonates with delayed transition when compared to TTN (Table 1).

**Table 1:** Clinical characteristics of neonates

	TTN (*n* = 21)	Delayed transition (*n* = 28)	Controls (*n* = 30)	*p*-value^a^
Birth Weight (gr)	3270 (3130–3460)	3580 (3285–3710)	3205 (2980–3350)	0.006
Gestational age (weeks)	38 (38–39)	39 (38–39)	38 (38–39)	0.031
Early-term n, (%)	14 (67)	13 (46)	18 (60)	NS
Male gender n, (%)	13 (61)	10 (36)	13 (43)	NS
Tachypnea duration (hours)	24 (18–32)	3 (2–3)	-	-

*NS* non-significant

^a^Kruskal-Wallis Test was performed for birth weight and gestational age between groups; statistical significance observed for birth weight; control vs delayed transition (*p* = 0.001) and gestational age; TTN vs delayed transition (*p* = 0.007)

All neonates had capillary refill time less than 3 s. Newborns’ HR, SpO2 and axillary temperature were similar between three groups. The RR at 10^th^ minute and 1^st^ hour were significantly higher in delayed transition and TTN groups when compared with controls (Table 2).

**Table 2:** Heart rate, respiratory rate, SpO2 and axillary temperature of neonates and controls

		TTN (*n* = 21)	Delayed transition (*n* = 28)	Controls (*n* = 30)	*p*-value^a^
Heart rate (beats/min)	10^th^ minute	148 (138–156)	149 (145–153)	152 (140–160)	NS
	1^st^ hour	148 (138–156)	145 (138–149)	143 (136–157)	NS
Respiratory rate (breaths/min)	10^th^ minute	72 (68–80)	72 (68–74)	56 (52–58)	*p* < 0.0001
	1^st^ hour	76 (72–82)	64 (58–68)	54 (50–56)	*p* < 0.0001
SpO2 (%)	10^th^ minute	97 (96–99)	98 (97–98)	98 (97–100)	NS
	1^st^ hour	98 (97–98)	98 (97–99)	98 (97–100)	NS
Axillary temperature	10^th^ minute	36.2 (36.0–36.3)	36.2 (36.0–36.4)	36.0 (36.0–36.2)	NS
	1^st^ hour	36.6 (36.5–36.6)	36.5 (36.5–36.7)	36.5 (36.4–36.7)	NS

*NS* non-significant, Values are expressed as median (IQR)

^a^Kruskal-Wallis Test; statistical significance observed for respiratory rate (10^th^ minute); control vs delayed transition, control vs TTN and for respiratory rate (1^st^ hour); control vs delayed transition, control vs TTN, and delayed transition vs TTN

**Fig. 1:**
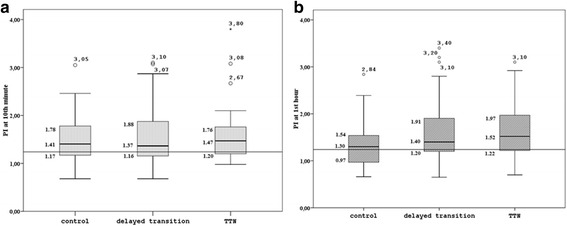
Perfusion index at 10^th^ minute (**a**) and 1^st^ hour (**b**) of control, delayed transition and TTN groups. The *p*-value is not significant for PI measurements between groups and paired measurements within groups. *Horizontal line* indicates the 1.24 PI value. Lower and upper margin of each *box* represents 25^th^ and 75^th^ percentiles, *horizontal lines* in the middle of the boxes represents median value, and whiskers represent 10^th^ and 90^th^ percentiles

The PI values were similar between three groups both at 10^th^ minute and at 1^st^ hour. We did not observe a significant change from 10^th^ minute to 1^st^ hour in each group as seen in Fig. 1.

Two babies’ both PI values were lower than 0.7; one was in the control group and the other was in the delayed transition group. Echocardiography excluded congenital heart disease in these neonates. There were seven neonates with both PI values between 0.7 and 1.0, eight neonates with either of two PI values between 0.7 and 1.0. None of those had TTN. In 26 neonates either one of the two PI values were lower than 1.24. The rates of PI lower than 1.24 among control, delayed transition and TTN groups were similar; (10^th^ minute measurements; 40 %, 29 %, 29 %; *p* = 941) and (1^st^ hour measurements; 30 %, 35 %, 33 %; *p* = 0.153).

When all paired PI, HR, RR and temperature data were pooled, no correlation existed between PI and RR, HR, and axillary temperature (Spearman’s correlation analysis; PI and RR; *p* = 1.189, PI and HR; *p* = 0.395, PI and temperature; *p* = 0.419). SpO2 was not included in correlation analyses as all values were above 92 %.

Neonates diagnosed as delayed transition received oxygen treatment only (7/28) during the observational period in the maternity unit. The neonates with TTN were treated with intravenous fluid administration (21/21), oxygen treatment (15/21), nasal noninvasive ventilation (8/21).

## Discussion

This study evaluated the postductal PI values at 10^th^ minute and 1^st^ hour following birth in early-term and full-term newborns. Our findings demonstrated that early postnatal PI values did not differentiate the newborns with delayed transition and those with TTN, confirming that postnatal PI is not a useful predictor of which newborns require admission to the NICU for TTN. It is known that a number hemodynamic changes occur in the newborn circulation right after birth. These changes include but are not limited to increased pulmonary blood flow, closure of intrauterine shunts, fluid shifts between intracellular and extracellular compartments, and clearance of lung fluid [22]. Increase of left ventricular output and decrease of proportion of right to left shunts are important components of this period [23, 24]. In some newborns these changes may occur less smoothly than others and in some babies may require NICU admission due to delayed adaptation to birth. The problems that may occur during the transitional period may necessitate respiratory and circulatory support on a wide spectrum. However it is not always easy to discriminate adaptation problems from those that are seen during sepsis, pneumonia or heart disease. Therefore several methods of assessment are attempted to differentiate normal adaptation from delayed transition and other diseases. Peripheral perfusion assessment has been considered to be potentially helpful for circulatory failure determination. We wanted to see whether peripheral PI actually was different in healthy newborns and in newborns with delayed transition or TTN. Both of these conditions theoretically are not expected to cause any overt circulatory problems however the microcirculatory pattern is not that clear. The fact that we did not differentiate neonates with delayed transition or controls from those with TTN by determining PI was in accordance with the accepted approach once again proving that TTN or delayed transition in otherwise healthy newborns does not affect peripheral perfusion. The similar PI in all groups might be due to the fact that the differences in the cardiopulmonary transition process among groups were not abundant to effect the PI assessments.

The first PI measurements (10^th^ minute) were referred to immediate transition at birth which did not differ between groups. Kroese et al. also investigated the PI values of term infants during the first 10 min of life and demonstrated that PI values were stable during the transition at birth [2.0–2.4 (1.3–5.0)]. Our results were found to be lower [1.41 (1.17–1.78)] than the authors’ at 10^th^ minute [2.0 (1.4–3.1)] [3]. The measurement site; postductal – preductal; may have caused this difference. We decided to measure postductal PI as it may provide a better information about the peripheral circulation than preductal measurements since the circulation changes after birth, and direction and proportion of shunts through foramen ovale and ductus arteriosus are mainly dependent on the pulmonary vascular resistance. This finding is compatible with the results of Hakan et al. which pointed that preductal PI values were higher than postductal PI values [25]. The distribution of PI values measured at 10th min after birth was more uniform in TTN patients compared to the two other groups, perhaps due to less activity and crying in this group. We have observed that PI values fluctuate considerably during crying and increased activity. Infants with TTN seemed to be less active compared to the other two groups most likely in an effort to preserve energy and consume less oxygen. The variations in PI measurements we observed were emphasized previously both in preterm and term infants [3, 4].

We evaluated the second PI measurements at 1^st^ postnatal hour of life, we did not observe either a change in PI over time or a difference between groups. The median PI values of groups were in-between the reported PI values in literature as 1.7 by Granelli and Ostman-Smith and 1.0 by Hakan et al. [21, 25].

De Felice et al. demonstrated that early low PI may be associated with histologic chorioamnionitis (histologic chorioamnionitis vs controls, 1^st^ minute; 1.74 vs 4.50 and 5^th^ minute; 2.18 vs 4.52) which is an important risk factor for neonatal morbidity. Though, in that study, admissions to NICU did not differ among neonates when grouped according to the cut-off values [6]. Their results were remarkable, however one interesting point is that; the PI values that the authors’ demonstrated were higher than ours and the literature. Our study was not designed to investigate placental pathology however undiagnosed histologic chorioamnionitis could not explain the low PI values in our study. We previously demonstrated slightly higher PI measurements in neonates born with C/S; 1.8 (0.5–5.0); compared to present study which was performed in similar environment and with the same monitor [5]. This shows that individual differences in maternal care, operation – anesthesia procedure or neonatal behavior may have considerable effect on results.

Some studies determined the cut-off values for certain neonatal conditions. Granelli and Ostman-Smith suggested that PI values lower than 0.70 may show left ventricle outflow obstruction, and PI values lower than 1.0 must be taken into consideration for duct dependent congenital heart diseases [21]. However we did not determine any congenital heart disease in neonates with PI lower than 1.0. Another cut-off value was demonstrated to be 1.24 in NICU patients as a predictor for severity of illness by De Felice et al. [20]. About %33 of included neonates in our study had either one of PI values below 1.24 and their proportion did not differ between groups. Our finding is consistent with study by Kroese et al., in which the authors reported 25 % of healthy neonates had PI value lower than 1.24 and none required medical care [3]. We think that this cut-off value should be approached with caution particularly for neonates in maternity unit and is not suitable to be used during transitional period.

We experienced high PI values up to 8.0 while baby was crying and observed the decline in values as a steady state was succeeded. Granelli and Ostman-Smith especially pointed that PI values greater than 4.5 may be the result of vasodilatation due to child screaming and must be repeated [21]. Hummler et al. pointed in their study that a low PI value was associated with increased risk of bias and low PI values were attributed to local vasospasm, hypothermia [26]. Those observations emphasized the importance of consideration of infants’ activity during PI assessment.

There are some limitations of our study. Firstly, we did not include the neonates with congenital pneumonia. As known, initial symptoms of infants with TTN and congenital pneumonia are indistinguishable and presence of maternal risk factors supports the diagnosis of pneumonia [27]. If there were a fourth group of neonates with pneumonia, PI values among TTN and congenital pneumonia could have also been evaluated which can be further investigated in future studies with a larger patient population. Another limitation is the small sample size which was decided by power analysis to detect a 30 % difference (one standard deviation) in PI values. A study designed to show significant difference of 10 % in PI values would require 163 patients in each group. Also our study was based on the PI values only during first 1 h. The PI values at 6^th^ hour life may be of value and could be investigated in further studies to see whether it is useful to discriminate TTN from the neonates with delayed transition.

## Conclusions

PI assessment in maternity unit does not discriminate neonates with TTN either from those with delayed transition or from healthy neonates. Furthermore we suggest that newborns with TTN do have lower PI values when compared to healthy newborns.

## Abbreviations

HR: Heart rate
NICU: Neonatal intensive care unit
PI: Perfusion index
RR: Respiratory rate
SpO2: Pulse oximetry oxygen saturation
TTN: Transient tachypnea of newborn
